# Does 3D Printing-Assisted Acetabular or Pelvic Fracture Surgery Shorten Hospitalization Durations among Older Adults?

**DOI:** 10.3390/jpm12020189

**Published:** 2022-01-31

**Authors:** Chun-Chi Hung, Jia-Lin Wu, Yung-Wen Cheng, Wei-Liang Chen, Shih-Han Lee, Tsu-Te Yeh

**Affiliations:** 1 Department of Orthopaedic Surgery, Tri-Service General Hospital, and School of Medicine, National Defense Medical Center, Taipei 114, Taiwan; raffi10110126@gmail.com; 2 Division of Traumatology, Department of Surgery, Tri-Service General Hospital, and School of Medicine, National Defense Medical Center, Taipei 114, Taiwan; 3 Department of Orthopedics, School of Medicine, College of Medicine, Taipei Medical University, Taipei 110, Taiwan; wu.jialin@tmu.edu.tw (J.-L.W.); kozuetora@gmail.com (S.-H.L.); 4 Department of Orthopedics, Taipei Medical University Hospital, Taipei 110, Taiwan; 5 Orthopedics Research Center, Taipei Medical University Hospital, Taipei 110, Taiwan; 6 Centers for Regional Anesthesia and Pain Medicine, Wan Fang Hospital, Taipei Medical University, Taipei 116, Taiwan; 7 Division of Family Medicine, Department of Family and Community Medicine, Tri-Service General Hospital, and School of Medicine, National Defense Medical Center, Taipei 114, Taiwan; cyw0451@gmail.com (Y.-W.C.); weiliang0508@gmail.com (W.-L.C.); 8 Division of Geriatric Medicine, Department of Family and Community Medicine, Tri-Service General Hospital, and School of Medicine, National Defense Medical Center, Taipei 114, Taiwan; 9 Department of Biochemistry, National Defense Medical Center, Taipei 114, Taiwan

**Keywords:** 3D printing-assisted surgery, pelvis fracture, acetabulum fracture, length of hospital stay, geriatric patient

## Abstract

Acetabular or anterior pelvic ring fractures are rare but extremely complicated and challenging injuries for orthopedic trauma surgeons. Three-dimensional (3D) printing technology is widely used in the management of these two fracture types for surgical benefits. Our study aimed to explore whether 3D printing-assisted acetabular or pelvic surgery is beneficial in terms of shortening the length of hospital stay (LHS) and intensive care unit (ICU) stay (ICU LS) for older patients. This retrospective study included two groups of 76 participants over 60 years old who underwent operations with (*n* = 41) or without (*n* = 35) guidance by 3D printing. The Mann–Whitney *U* test was used to analyze continuous variables. Chi-square analysis was applied for categorical variables. Univariable and multivariable linear regression models were used to analyze the factors associated with LHS. The median LHS in the group without 3D printing assistance was 16 (12–21) days, and the median ICU LS was 0 (0–2) days. The median LHS in the group with 3D printing assistance was 17 (12.5–22.5) days, and the median ICU LS was 0 (0–3) days. There was no significant difference in LHS associated with 3D printing assistance vs. that without 3D printing among patients who underwent open reduction and internal fixation for pelvic or acetabular fractures. The LHS positively correlated with the ICU LS whether the operation was 3D printing assisted or not. For fracture surgery in older patients, in addition to the advancement of surgical treatment and techniques, medical teams require more detailed preoperative evaluations, and more personalized medical plans regarding postoperative care to achieve the goals of shortening LHS, reducing healthcare costs, and reducing complication rates.

## 1. Introduction

Acetabular fractures are fractures of the hip-joint socket. In young people, acetabular fractures are mainly caused by high-energy trauma secondary to traffic accidents or falls from height and are often associated with other life-threatening injuries. Acetabular fractures sustained by older adults often result from minimal trauma, such as that associated with low falls. Acetabular fractures account for about 3% of all adult fractures [1,2]. These fractures are associated with serious morbidity and mortality [3]. Such fractures can be managed conservatively or surgically. Operative indications of acetabular fractures include displacements that involve the weight-bearing dome or cause femoral head incongruity and unstable posterior wall acetabular fractures [4]. The goal of surgery is to accurately restore the anatomic configuration of the joint’s surface, as well as the congruence and stability of the hip joint while avoiding complications. Surgical intervention of acetabular fractures has not been as successful in older patients as in younger patients. In comparing patients who are younger and older than 40 years of age, anatomic reductions were achieved in less than 60% of older patients, compared with nearly 80% of younger patients. In functional outcomes, younger patients had better clinical results of pain control, ambulatory ability, and hip range of motion than patients older than 40 years of age (81% vs. 68%) [5]. 

Pelvic fractures are fractures of the pelvic ring, including any breaks in the sacrum and the two innominate bones joined anteriorly at the symphysis and posteriorly at the paired sacroiliac joints. In younger patients, fractures of the pelvis, such as fractures of the acetabulum, also occur primarily as a result of high-velocity trauma. The incidence of pelvic ring injuries was reported to be 0.82 per 100,000 people and accounted for only 2% of all fractures [6]. The geriatric population constitutes about 22% of the overall number of patients with pelvic ring injuries. Men are affected slightly more than women (56% vs. 44%) [7]. Hemodynamic instability in patients with a pelvic ring fracture is a concern, and more aggressive means of addressing pelvic bleeding such as retroperitoneal packing and the continued use of angiography are being employed [8]. With the increasing age of the population and the unfortunate decreasing bone quality that accompanies aging, new fixation techniques are being evaluated and used in an effort to improve outcomes in the geriatric patient population [9]. The following situations are amenable to nonsurgical management: stable pelvic ring injuries, stable sacral injuries, comorbidities precluding surgical intervention, poor bone quality, and low-energy osteoporotic pelvic ring fracture. The appropriate treatment of pelvic injuries depends largely on the careful analysis of the fracture pattern, neurologic involvement, and associated pelvic ring disruption. The application of fixation implants is based on fracture patterns and orthopedic surgeons’ familiarity. Compared with other fractures, acetabular and pelvic fractures are more difficult to manage because of the complex anatomic structures in the surrounding area; these affect operation times, the amount of bleeding, and the patient’s functional recovery after healing [10].

Recent technologies in 3D printing have improved and expanded applications in many fields, such as engineering, medicine, architecture, and art and design. In many medical fields, creating tissues and organoids, surgical tools, patient-specific surgical models, and custom-made prosthetics are four main developments that are associated with recent innovations in 3D printing technology [11]. The application of 3D printing techniques to guide fracture surgery was first developed in the mid-1990s and has advanced significantly in recent years [12]. Three-dimensional printing techniques are widely applied in transplant, cardiovascular, orthopedic, and otolaryngologic surgery. Three-dimensional-printing-assisted fracture surgery is widely implemented for various complex fractures, such as pelvic fractures, acetabular fractures, and complex comminuted fractures [13]. Surgical technology—from preoperative fracture model analysis, to pre-contour steel plate application, to the design of the wound approach method—can provide surgeons with a more complete preoperative plan. Studies have found that 3D printing-assisted methods can effectively minimize operation times, wound size, blood loss, and postoperative recovery times, as well as improve functional scores [14,15]. To our knowledge, there is currently no relevant literature exploring whether 3D printing-assisted surgical treatment can shorten the length of hospital stay (LHS) and intensive care unit length of stay (ICU LS).

For older adults, LHS and ICU LS are associated with the rates of complications of hospitalization, such as delirium and nosocomial infections. Our study included patients over 60 years old who underwent surgical intervention for acetabular or pelvic fractures. We aimed to determine whether 3D printing-assisted surgery could shorten the LHS and the ICU LS.

## 2. Materials and Methods

### 2.1. Study Design and Participant Selection

This was a retrospective study that assessed participants with acetabular or pelvic fractures who underwent surgical treatment at the Tri-Service General Hospital in Taiwan from August 2009 to December 2021. We included participants (1) aged ≥ 60 years, (2) with acetabular or pelvic fractures diagnosed by computed tomography (CT), and (3) who underwent open reduction and internal fixation (ORIF) with plates or spinal–pelvic instructs. Patients who died and those who had complications of ventilator dependency or minimally invasive screw fixation surgery for anterior column fracture and sacroiliac joint injuries were excluded. The patients included in the study were divided into two groups: those who underwent 3D printing-assisted surgery and those who did not. The study was approved by the Tri-Service General Hospital Institutional Review Board (TSGH IRB). All study participants gave written informed consent, and the study was conducted in accordance with the provisions of the Declaration of Helsinki.

### 2.2. Surgical Treatment

All operations were performed with the participants under general anesthesia by a single experienced orthopedist. According to the surgical approach, the patient was placed on a radiotransparent operating table in a supine or lateral decubitus position. For acetabulum fractures, the Kocher–Langenbeck approach was applied for a posterior wall fracture or posterior column fracture. The ilioinguinal approach or the modified Stoppa approach was applied for an anterior column fracture. For pelvic fractures, the anterior or posterior approach was used. Depending on the fracture pattern, a one-stage or two-stage procedure was performed.

In the group without 3D printing, the reconstruction plate was contoured according to the template determining the length and shape. The plate-bending instrument was applied for minor adjustment. In the group with 3D printing, CT scanning images were converted to generate patient-specific 3D printing models (1:1 models). The required plate length, position, and location, were determined by this patient-specific 3D-printed model. The straight locking reconstruction plate was contoured preoperatively according to this model. The 3D printing technique was applied for the treatment of anterior pelvic ring and acetabular fractures; otherwise, the posterior pelvic ring fractures in both groups were managed with traditional surgical methods. 

**Figure 1:**
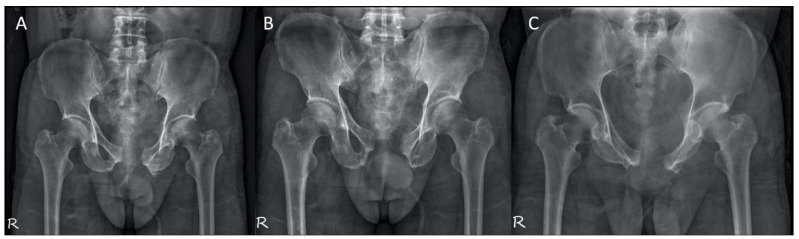
Preoperative X-ray films of the pelvis. (**A**) AP view, (**B**) outlet view, and (**C**) inlet view.

**Figure 2:**
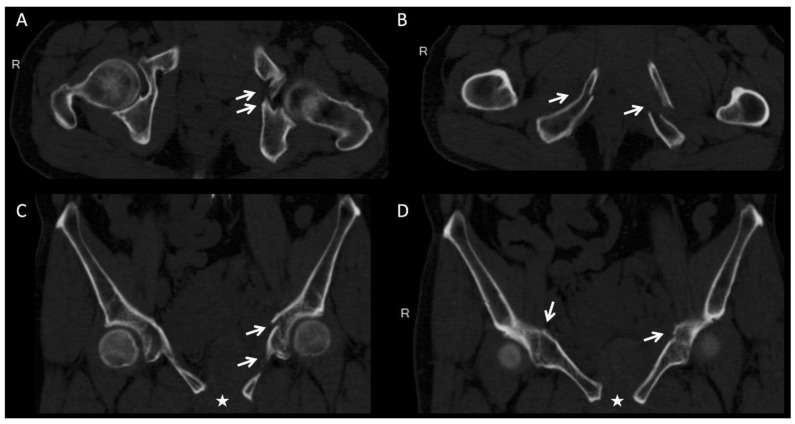
Computed tomography scan images showing bilateral superior and inferior pubic ramus fracture (arrows) combined with diastasis of pubic symphysis (asterisks). (**A**,**B**) Axial view and (**C**,**D**) coronal view.

**Figure 3:**
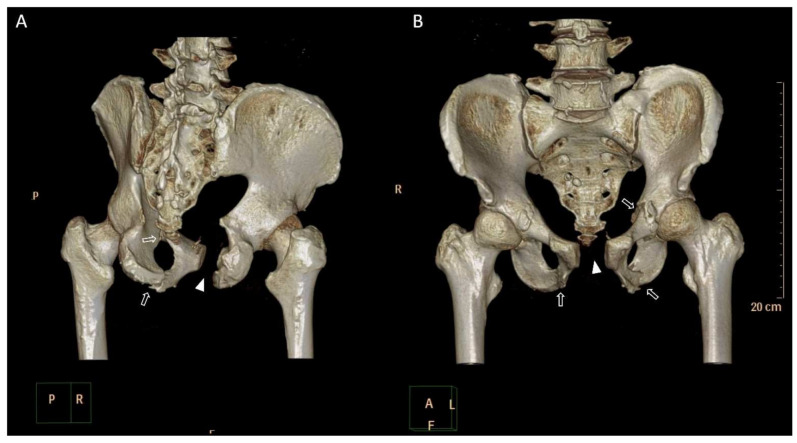
Three-dimensional reconstruction computed tomography scan images showing bilateral superior and inferior pubic ramus fracture (hollow arrows) combined with diastasis of pubic symphysis (triangles) from (**A**) posterior aspect and (**B**) anterior aspect.

**Figure 4:**
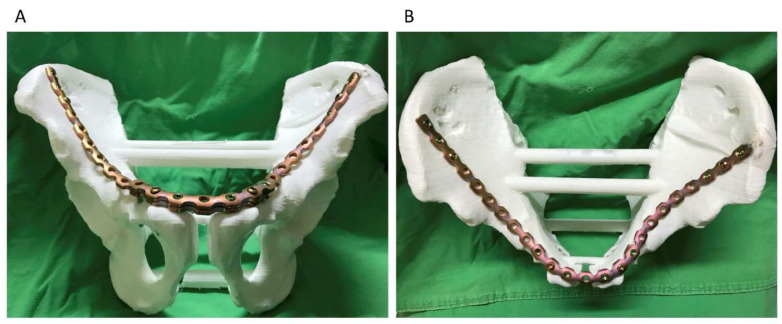
Three-dimensional printing model of reduced bilateral iliac wing and pre-contoured plates in overlapping configuration. (**A**) Anterior view and (**B**) Superior view.

**Figure 5:**
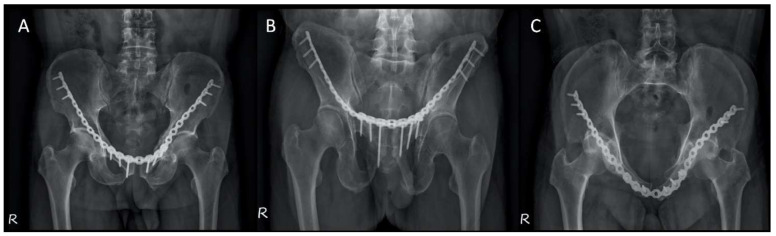
Postoperative X-ray films of the pelvis. (**A**) AP view, (**B**) outlet view, and (**C**) inlet view.

We applied the 3D printing-assisted technique to the treatment of a 61-year-old man with a diagnosis of bilateral superior and inferior pubic ramus fracture combined with diastasis of pubic symphysis.. The patient received pelvic X-ray exams initially (Figure 1). He underwent diagnostic CT scans to confirm the fracture sites (Figure 2). The CT scan images were converted to DICOM images and reconstruction images (Figure 3). To create the reduced fracture model, the techniques of segmentation, splitting, mirroring, and reposition were conducted through MIMICS software (version 19, Belgium), and exported as a 3D model. The pre-contour plates were manufactured according to this customized model preoperatively (Figure 4). The postoperative X-rays are presented in Figure 5. Anatomical reduction of the fracture was achieved, which requires more time and expertise on the part of the surgeon in complex pelvic surgeries.

### 2.3. Covariates

Age, gender, and body mass index were obtained from the medical records. Laboratory data were obtained from preoperative evaluation data, including hemoglobin, platelets, serum glucose, creatinine, aspartate transaminase (AST), sodium, and potassium levels. Surgical subgroups were analyzed based on a combination of other types of major trauma, as detailed in the medical records of the emergency department or outpatient clinic: fracture other than pelvis or acetabulum, brain hemorrhage, pneumo/hemothorax, internal organ hemorrhage, and vessel rupture requiring transarterial embolization. Operation types were categorized as pelvic fracture, acetabular fracture, combination of both pelvic and acetabular fractures from the same traumatic event, and pathological fractures were defined as those due to bone tumors, including primary or metastatic bone tumors. Based on the surgical plans of a single experienced operator, the procedures were categorized as a one-stage surgery or a two-stage surgery. 

### 2.4. Statistical Analysis

Continuous variables are expressed as medians and interquartile ranges (IQR, Q1–Q3). Categorical variables are expressed as numbers and percentages (%). Statistical significance was analyzed using the Mann–Whitney *U* test for continuous variables and chi-square tests for categorical variables using PASW Statistics for Windows, version 18.0 (SPSS Inc., Chicago, IL, USA). The Mann–Whitney *U* test was used for the subgroup analysis of patients with or without a combination of other major trauma in the hospital and ICU stay course. Simple linear regression modeling was used to analyze the regression coefficients of LHS on the univariable factors among participants with and without 3D printing-assisted operations. The factors with *p*-values < 0.1 in the univariable linear regression underwent multivariable linear regression. Statistically significant differences were defined by *p* values < 0.05.

## 3. Results

### 3.1. Characteristics of the Study Participants

A total of 76 consecutive patients were included and divided into groups of patients whose operations did not rely on 3D printing (*n* = 35) and patients whose operations were guided by 3D printing (*n* = 41). Table 1 demonstrates the patients’ demographic characteristics. In the group without 3D printing assistance, there were 16 men and 19 women, and the median age was 65 (61–73) years. In the group with 3D printing assistance, there were 17 men and 24 women, and the median age was 67 (62–74.5) years. Sixteen (45.7%) participants had other major trauma in the group without 3D printing assistance, and 12 (29.3%) had other major trauma in the group with 3D printing assistance. The median LHS for the group without 3D printing assistance was 16 (12–21) days, and the median ICU LS was 0 (0–2) days. The median LHS for the group with 3D printing assistance was 17 (12.5–22.5) days, and the median ICU LS was 0 (0–3) days (all *p*-values > 0.05).

### 3.2. Measurable Factors and the LHS

Table 2 summarizes the subgroup analysis of combinations of other major traumas. There were no significant differences in the LHS and ICU LS between the subgroups with or without combinations of other traumas. Table 3 demonstrates the gender subgroups analysis. There were no significant differences in the LHS and ICU LS between males and females (all *p*-value > 0.05). The regression coefficients of LHS on the measurable factors among patients whose operations were not guided by 3D printing are detailed in Table 4. LHS was negatively correlated with platelet count (β, *p*-value: −0.055, 0.019) and positively correlated with ICU LS (β, *p*-value: 1.685, 0.004) in the multivariable regression model. The regression coefficients of LHS on the measurable factors of patients who underwent 3D printing-assisted operations are detailed in Table 5. ICU LS was positively correlated with LHS (β, *p*-value: 1.131, 0.035), and preoperative serum potassium level was negatively correlated with LHS in the univariable regression models (β, *p*-value: −4.934, 0.022). In the multivariable regression model, LHS was positively correlated with ICU LS and negatively correlated with serum potassium level (β, *p*-value: −4.934, 0.022).

## 4. Discussion

To our knowledge, our study is the first to explore the impact of 3D printing on the LHS for patients older than 60 years of age who underwent surgery for pelvic and acetabular fractures. We found no significant difference in the LHS and the ICU LS between the groups with and without 3D-assisted surgery. We applied multivariable regression to analyze the correlation between the parameters of preoperative evaluation and LHS in both groups. The LHS was significantly and positively associated with the ICU LS in both groups. In the group without 3D printing assistance, the LHS was negatively associated with preoperative platelet levels. In the 3D printing-assisted group, the LHS was negatively associated with preoperative serum potassium levels.

With the aid of 3D printing for complex fractures, operation times can be effectively minimized, along with intraoperative blood loss and surgical wound size [14,15,16]. Typically, pelvic fractures require longer operation times than other types of fractures. The complexity of the surrounding structures also increases the difficulty of the operation [17,18,19,20]. The longer the operation time, the higher the risks of anesthesia- and surgery-related complications. In the application of 3D printing techniques, there has been great progress and increased use in the past 10 years [21,22,23]. Our team previously published research on 3D printing-technology-assisted pelvic fracture surgery, which can shorten operation times and reduce the amount of bleeding. Moreover, it has significant benefits for surgical efficacy and postoperative rehabilitation [14,15].

LHS is also an important consideration for the care of patients. At present, many improvements in surgical methods, the evolution of different bone-fixation materials, and various dressings, for example, are also aimed at reducing the complications of surgery and shortening ICU LS and overall LHS. Prolonged hospitalization increases the rates of nosocomial infections and delirium, hospital costs, and functional decline [24,25,26,27]. Our research found no significant effect of 3D printing-assisted surgery on LHS. A possible explanation is that the initial wound-healing process of older patients takes longer than that of younger patients [28,29]. Older adults also have more comorbidities^17^. During hospitalization, acute physiological stress causes changes in preexisting chronic diseases, such as diabetes mellitus and hypertension, which affects the safety considerations for assessing and discharging patients [30,31,32,33,34]. These factors are encountered regardless of whether 3D printing techniques are used or not.

Taiwan has a universal health insurance system, and patients and their families hope that they can be taken care of before being discharged from the hospital in a relatively stable condition [35,36]. Whether patients with pelvic fractures can be discharged from the hospital is mainly evaluated by the attending physician and medical team. In principle, if the surgical wound is adequately healed, patients without other complications can be discharged from the hospital if they are in stable condition. The time to discharge patients from the hospital depends on the medical team’s discretion, the condition of patients, and sometimes the caregiver’s or family’s expectations, and the LHS results of the two groups may be similar because of these multifactorial contributions.

The ICU LS depends mainly on the initial injury of the patient and whether the patient is at high risk. It is necessary to conduct close monitoring in the ICU for postoperative and post-anesthesia care. This is jointly managed by the surgeon and the anesthesiologist. In the case of multiple traumas, particularly in combination with changes in consciousness and other major organ trauma, including cerebral hemorrhage, pneumo/hemothorax, or other organ bleeding caused by hemodynamic imbalance, and the use of ventilators, ICU LS increases [37]. This may explain the lack of a statistical difference between the two groups we compared. In Table 2, we provide a subgroup analysis of the presence or absence of trauma in other organ systems. For patients with pelvic or acetabular fractures, there was no statistical difference in the number of hospitalization days and ICU days whether combined with other major trauma or not. 

To investigate specific factors associated with LHS and ICU LS, we collected preoperative biomarker data. We found that a lower preoperative potassium concentration was associated with a longer ICU LS. A low potassium level may indicate electrolyte imbalance. More serious trauma, or a frailer pre-injury state, along with electrolyte imbalances and poor nutritional status, for example, may be risk factors that lengthen hospital stays. In the regression model, ICU LS was positively correlated with LHS. Before the development of 3D printing, because of the higher risks associated with surgery, risk of blood loss, and the high technical threshold of pelvic and acetabular surgery, a higher proportion of patients with pelvic fractures in the past chose conservative treatment. Thus, the average LHS and ICU LS of the group without 3D printing assistance may have been underestimated. Preoperative investigations before and during admission do have predictable effects on LHS, including in terms of early mortality after open abdominal surgery [38]. The severity of electrolyte imbalances has been associated with longer LHS and increased in-hospital, 30-day, and 1-year mortality in an unselected emergency department population [39]. Preoperative low and high platelet counts have been correlated with perioperative complications after total hip arthroplasty, including major or minor adverse effects and hospital readmissions [40]. 

The present study has some limitations. First, this was a retrospective study. Due to the rarity of pelvic or acetabular fractures, the sample size was small. We did not perform a sample size calculation, and the limited number of study patients may have affected the statistical significance of our results. Confounding factors related to the patients and interventions also exist. Second, the past medical history, nutritional status, daily functional status, and the bone health of participants are variable, and limited data regarding these variables could be obtained from the medical records. Third, the indicator that we used to assess the risk of anesthesia and surgery was ICU LS (whether or not the patient required postoperative ICU admission), and the length of stay in the ICU was jointly determined by the surgeon and the anesthesiologist. Larger-scale, prospective studies are required to further evaluate the clinical applications of 3D printing-assisted technology for treating pelvic and acetabular fractures. Moreover, more rigorous study designs (e.g., randomized controlled trials with blinding) should be considered in the future.

## 5. Conclusions

To our knowledge, our study is the first to determine the lack of a significant difference in LHS among older pelvic or acetabular fracture patients receiving ORIF with or without 3D printing assistance. Current evidence suggests that 3D printing-assisted surgery helps improve intraoperative parameters, but it may not affect the patient’s postoperative recovery. For fracture surgery for older patients, in addition to the advancement of surgical treatment and techniques, medical teams require more detailed preoperative evaluations, and more personalized medical plans regarding postoperative care to achieve the goals of shortening LHS, reducing healthcare costs, and reducing complication rates.

## Figures and Tables

**Table 1:** Demographics.

	Without 3D (*n* = 35)	With 3D (*n* = 41)	*p* Value *
Category variable, *n* (%)			
Sex			0.709
Man	16 (45.7)	17 (41.5)	
Woman	19 (54.3)	24 (58.5)	
Combined with other major trauma			0.138
with	16 (45.7)	12 (29.3)	
without	19 (54.3)	29 (70.7)	
Operation type			0.137
Pelvis	17 (48.6)	18 (43.9)	
Acetabulum	12 (34.3)	14 (34.1)	
Both	5 (14.3)	2 (4.9)	
Pathological fracture	1 (2.9)	7 (17.1)	
Surgical stage			0.425
One-stage	26 (74.3)	27 (65.9)	
Two-stage	9 (25.7)	14 (34.1)	
Continuous variables, median (IQR)			
Age, year	65 (61–73)	67 (62–74.5)	0.155
Hb, g/dL	12.5 (10.7–13.5)	11.2 (10.1–12.7)	0.057
Platelet, 10^3^/uL	192 (151–233)	221 (160–306.5)	0.067
Glucose, mg/dL	130 (106–155)	130 (112–169.5)	0.381
Creatinine, mg/dL	0.8 (0.6–1.0)	0.8 (0.7–1.15)	0.185
AST, U/L	29 (20–40)	27 (19–32.5)	0.287
Sodium, mmol/L	138 (136–139)	137 (136–139.5)	0.937
Potassium, mmol/L	3.8 (3.6–4.1)	3.9 (3.75–4.3)	0.114
BMI, kg/m^2^	23.1 (21.6–26.8)	22.9 (20.5–25.8)	0.402
Length of hospital stay, day	16 (12–21)	17 (12.5–22.5)	0.531
Length of stay in ICU, day	0 (0–2)	0 (0–3)	0.432

* Chi-square test for category variables and Mann–Whitney *U* test for continuous variables. Abbreviations: Hb, hemoglobin; AST, aspartate aminotransferase; BMI, body mass index.

**Table 2:** Subgroup analysis of combination with other major trauma (median, IQR).

	Without 3D (*n* = 35)	With 3D (*n* = 41)	*p* Value *
Without other major trauma	*n* = 19	*n* = 29	
Length of hospital stay	15 (11–19)	16 (12–22.5)	0.245
Length of stay in ICU	0 (0–2)	0 (0–2)	0.381
With other major trauma	*n* = 16	*n* = 12	
Length of hospital stay	18 (14.5–24.75)	19.5 (15.25–22.75)	0.945
Length of stay in ICU	0 (0–2)	1 (0–4)	0.767

* Mann-Whitney *U* Test.

**Table 3:** Subgroup analysis of sex (median, IQR).

	Without 3D (*n* = 35)	With 3D (*n* = 41)	*p* Value *
Male	*n* = 16	*n* = 17	
Length of hospital stay	17 (12.25–21)	16 (12–24)	0.709
Length of stay in ICU	1 (0–3)	0 (0–3)	0.736
Female	*n* = 19	*n* = 24	
Length of hospital stay	16 (11–21)	17.5 (13.25–22)	0.651
Length of stay in ICU	0 (0–2)	0 (1–3)	0.127

* Mann–Whitney *U* Test.

**Table 4:** Regression coefficients of length of hospital stay on the measurable factors in participants without 3D-assisted operation.

	Univariable ^1^		Multivariable ^2^	
	β (SE)	*p* Value	β (SE)	*p* Value
Sex	−0.579 (3.320)	0.863		
Combined with other major trauma	3.451 (3.266)	0.298		
Operation type	−0.469 (2.003)	0.819		
Surgical stage	2.868 (3.752)	0.450		
Age	0.152 (0.257)	0.558		
Hb	−1.017 (0.829)	0.229		
Platelet	−0.060 (0.025)	0.021	−0.055 (0.022)	0.019
Glucose	0.039 (0.051)	0.446		
Creatinine	−1.322 (6.273)	0.834		
AST	0.008 (0.029)	0.777		
Na	0.309 (0.763)	0.688		
K	−2.256 (4.278)	0.601		
BMI	−0.075 (0.384)	0.847		
Length of stay in ICU	1.790 (0.581)	0.004	1.685 (0.543)	0.004

^1^: univariable linear regression. ^2^: multivariable linear regression. Abbreviations: Hb, hemoglobin; AST, aspartate aminotransferase; BMI, body mass index; ICU, intensive care unit.

**Table 5:** Regression coefficients of Length of hospital stay on the measurable factors in participants with 3D-assisted operation.

	Univariable ^1^		Multivariable ^2^	
	β (SE)	*p* Value	β (SE)	*p* Value
Sex	1.586 (2.216)	0.479		
Combined with other major trauma	1.486 (2.404)	0.540		
Operation type	0.036 (1.017)	0.972		
Surgical stage	4.542 (2.201)	0.046	2.896 (2.052)	0.167
Age	0.028 (0.136)	0.836		
Hb	0.240 (0.537)	0.658		
Platelet	−0.006 (0.008)	0.480		
Glucose	0.004 (0.021)	0.848		
Creatinine	−0.140 (0.493)	0.778		
AST	−0.116 (0.089)	0.202		
Na	−0.154 (0.270)	0.571		
K	−6.104 (2.108)	0.006	−4.934 (2.060)	0.022
BMI	−0.140	0.661		
Length of stay in ICU	1.211 (0.566)	0.026	1.131 (0.517)	0.035

^1^: univariable linear regression. ^2^: multivariable linear regression. Abbreviations: Hb, hemoglobin; AST, aspartate aminotransferase; BMI, body mass index; ICU, intensive care unit.

## Data Availability

Data is contained within the article or supplementary material.
